# Prolificacy-survival trade-off in Morada Nova sheep under Brazilian semiarid conditions

**DOI:** 10.1007/s11250-026-05087-6

**Published:** 2026-06-04

**Authors:** Robson Mateus Freitas Silveira, Aysllan Harlley Rodrigues Pereira, Mirela Balistrieri, Paula Renata Cortat, Kleibe de Moraes Silva, Eula Regina Carrara, Concepta Mcmanus, Aline Vieira Landim

**Affiliations:** 1 Present Address: Department of Animal Science, “Luiz de Queiroz” College of Agriculture, University of São Paulo (USP), Piracicaba, São Paulo, 13418900 Brazil; 2 Department of Animal Science, State University of Acaraú Valley (UVA), Sobral, Ceará 62040-370 Brazil; 3 Embrapa Caprinos e Ovinos, Sobral, Ceará Brazil; 4 Animal Science Institute, Beef Cattle Research Center, Sertãozinho, São Paulo, Brazil; 5 Center for Nuclear Energy in Agriculture, University of São Paulo (USP), São Paulo, Piracicaba, 13.416-000 Brazil

**Keywords:** Productive efficiency, Reproductive efficiency, Life-history trade-off, Semiarid environments, Local breeds

## Abstract

We used long-term data from 13 Morada Nova sheep flocks monitored between 1999 and 2015 in the Brazilian semi-arid region to evaluate how prolificacy and seasonality shape maternal reproductive output and progeny survival. For each ewe lambing event, we quantified total progeny weight at birth (PWB) and weaning (PWW), as well as survival rates at birth (PSRB) and weaning (PSRW), and classified births as occurring in the dry or rainy season. Ewes with single births showed lower PWB and PWW, but higher PSRB and PSRW, than ewes with multiple births (*p* < 0.0001). When the number of lambs at birth was included as a covariate, each additional lamb was associated with an increase of 1.74 kg in PWB and 3.51 kg in PWW (*p* < 0.0001), but also with an absolute reduction of 6% points in PSRB and 17% points in PSRW, findings that are consistent with a trade-off between litter size and offspring survival. Ewes with multiple births were more productive in terms of total kilograms of lamb weaned, despite lower survival rates per offspring. No significant association was found between type of birth and progeny across seasons (χ² test: *p* > 0.05). However, the number of lambs weaned differed slightly between seasons (*p* = 0.014), with marginally higher values in the dry period (1.65) than in the rainy period (1.60), although the magnitude of this difference was small. In Morada Nova ewes, multiple births increased total weaned lamb biomass but were associated with reduced progeny survival, indicating a trade-off between prolificacy and lamb viability. Intermediate prolificacy provided a more balanced compromise between productivity and survival under semiarid conditions.

## Introduction

The Morada Nova breed, native to in the northeastern region of Brazil, particularly in the municipality of Morada Nova, Ceará-is characterized by its small size, predominantly polled phenotype, and high prolificacy (Facó, 2008; Façanha et al. 2021). These animals are well adapted to semiarid conditions. Even under thermal stress and prolonged drought, they can produce desirable meat and hide traits. Studies report acceptable carcass yields and favorable meat quality attributes (e.g., pH, tenderness, cooking loss) as well as resilient skin characteristics, supporting their use for meat and leather production in harsh environments (Jacinto et al. 2004; Silveira et al. 2024; Alves et al. 2023). Accordingly, the Morada Nova breed represents a crucial genetic resource for sheep production in semiarid regions, where harsh climatic conditions can severely impact reproductive performance (Landim et al. 2022; Pereira et al. 2023).

Improving productivity is one of the primary strategies for increasing profitability in commercial sheep farming. Systems that achieve better production indicators are generally more profitable and sustainable (Silveira et al. 2021). In sheep production systems operating under environmental constraints, productivity gains are closely linked to improvements in reproductive efficiency and early-life performance of lambs. In this context, adapting livestock production systems to environmental constraints poses a major challenge to farmers and professionals in the field. One of the main difficulties in sheep production is the need to produce lamb earlier and at lower cost (Raineri et al. 2015).

Productive efficiency refers to the performance of ewes within the production system, reflecting their potential under specific nutritional and health management conditions (Ungerfeld, Rodriguez, and Perez-Clariget, 2022). Seasonal variation in reproductive behavior among sheep breeds is influenced by evolutionary adaptations to their native environments. For instance, Soay ewes may experience only one to three estrous cycles per year (Grubb and Jewell 1973), while many hair sheep breeds (e.g., Santa Inês and Dorper) show only minor seasonal variation in reproductive activity (Rodrigues et al. 2007; Soares et al. 2015). Thus, regional environmental conditions play a critical role in shaping reproductive management strategies across different breeds.

Key traits such as lamb weight at weaning significantly impact the economic viability of sheep production, as they are closely tied to the system’s capacity to meet consumer demand (Silveira et al. 2021). Maternal traits, including prolificacy and maternal ability, are essential. While high prolificacy increases the number of lambs per parturition, it does not necessarily ensure their survival, which also depends on maternal behavior and milk yield (Wilson et al. 2025). This relationship exemplifies a classic *life-history trade-off*, in which increased reproductive output may be offset by reduced investment per offspring, potentially compromising survival. Therefore, it is crucial that ewes not only produce multiple offspring but are also capable of raising them successfully until weaning, thereby enhancing their overall productive efficiency by balancing reproductive quantity and offspring survival within this life-history framework.

Although several studies have addressed the impact of prolificacy, lamb weight, and survival in breeds such as Merino (Haslin et al. 2023; Lockwood et al. 2023), Dorset, Hampshire, and Suffolk (Osorio-Avalos et al. 2012), data on these aspects are scarce for Morada Nova sheep; despite their adaptation to the harsh conditions of northeastern Brazil and their economic importance, Morada Nova ewes remain underrepresented in the scientific literature, and no large-scale analysis has quantified how prolificacy affects lamb survival in this breed. We expected prolific ewes to have lower lamb survival but higher total lamb output.

This study aimed to evaluate the effects of prolificacy and seasonality on the performance of Morada Nova ewes by analyzing the total weight of offspring per ewe at birth and at weaning, as well as lamb survival at these stages, and to evaluate whether lambs born in the dry season differ in weight from those born in the rainy season.

## Materials and methods

### Ethics statement

All procedures involving animals were reviewed and approved by the Ethics Committee on the Use of Animals (CEUA) of Embrapa Goats and Sheep under protocol n^o^. 006/2016 and were conducted in accordance with the approved methodology, ensuring animal welfare.

#### Flock information

Thirteen Morada Nova sheep flocks located in different municipalities in CE, assessed between 1999 and 2015 were evaluated. The region is situated in the Caatinga biome, which is characterized by xerophytic vegetation composed predominantly of deciduous shrubs and small trees adapted to prolonged water deficit, irregular rainfall, and high temperatures. The climate is classified as BShw’ according to the Köppen classification (Alvares et al. 2013), typical of the Brazilian semi-arid region, and is characterized by a well-defined dry season (July to December) and a rainy season (January to June) (Araújo Filho et al. 2007; Vasconcelos et al. 2021). Annual precipitation in the region ranges from approximately 150 to 1,300 mm, with an average of about 700 mm, while mean air temperature is around 28 °C, varying between 18 °C and 40 °C. The average relative humidity is approximately 60%.

The dataset comprised 2,278 records from 779 unique ewes. Individual ewes contributed repeated measurements across successive parities, resulting in a hierarchical data structure with observations nested within animals (Table 1). The information regarding the flocks was managed and stored in the Flock Management System - SGR (Lôbo 2013). The SGR is a network-based software that allows the recording, storage, and management of information generated in goat and sheep herds, including zootechnical, reproductive, productive, sanitary, and pedigree data, enabling individual animal monitoring and supporting decision-making in herd management.

**Table 1:** Distribution of parturitions by type and year in Morada Nova ewes used in the study

Factors	Variables	*n*
Type of parturition	Simple	1252
	Double	921
	Triple	105
	Total	2278
Years	1999	38
	2000	44
	2001	47
	2002	82
	2003	39
	2004	34
	2005	114
	2006	150
	2007	223
	2008	263
	2009	323
	2010	338
	2011	197
	2012	225
	2013	151
	2014	5
	2015	5
	Total	2278

Animals were managed under a semi-extensive system, characterized by grazing on native rangeland with land allocation ranging from 1 to 3.0 ha per adult sheep, depending on seasonal forage availability, which is consistent with stocking rates commonly reported for small-ruminant production systems in semiarid regions. All lambs were raised with their dams until weaning, initially fed on maternal milk and subsequently grazing native pasture. Throughout the experimental period, animals had *ad libitum* access to water. In addition, all herds were managed under the same feeding regime and received concentrate supplementation based on ground corn and soybean meal, supplemented with a commercial vitamin-mineral premix. The level of concentrate supplementation varied according to the physiological stage of the ewes (lactating or dry), following nutritional recommendations for small ruminants established by the NRC (2007). Periods of greater pasture scarcity were defined based on seasonal reductions in forage availability and quality, typical of the semiarid production system, during which concentrate supplementation was routinely increased to meet nutritional requirements. Importantly, this feeding management was uniformly applied across all herds, minimizing potential nutritional confounding effects.

During the pre-weaning period, ewes grazed native pasture during the day, while lambs were kept in the facilities and had access to their dams for suckling. This management was adopted during the first 60 days of life, ensuring controlled maternal contact and protection of the lambs. After this period, lambs began to accompany their dams to native pasture, gradually increasing their exposure to grazing conditions. This management strategy allowed a progressive transition from exclusive milk-based nutrition to pasture-based feeding, without imposing a fixed or abrupt weaning age.

Sanitary management included monitoring newborn lambs, ensuring colostrum ingestion, and checking navel healing. Vermifugation was performed after the rainy season and as needed, following the recommendations for each animal category. During the final third of the gestation and at 15 days of age for the lambs, vaccinations against rabies and to control clostridiosis were performed. Parasite control was managed using the FAMACHA method (Bath et al. 1996; Van Wyk et al., 2002), with treatments every 15 days during the rainy season and every 30 days in the dry season. Additional sanitary control included monitoring for eimeriosis, also known as coccidiosis, which was treated with sulfonamides according to the manufacturer’s guidelines. After parturition, lambs’ navels were disinfected, and when excessively long, the umbilical cord was trimmed only after hemostasis, following standard veterinary procedures.

#### Characteristics studied

The characteristics associated with the productive efficiency of the females included total weight of lambs born and weaned per female per parturition (PWB and PWW, respectively), and the survival rate of the lambs at birth (PSRB) and at weaning (PSRW). Lambs were weighed at birth and weaning using a calibrated electronic scale to ensure measurement accuracy. The PWB was calculated by summing the weight of lambs born from the same birth per ewe. The PWW was determined by totaling the weights of the lambs at weaning, immediately after physical separation from the dam, for each ewe per parturition. The PSRB was obtained by dividing the number of alive and viable lambs at birth by the total number of lambs born per ewe per parturition. Similarly, PSRW was determined by dividing the number of lambs weaned by the total number of lambs born per ewe per parturition.

Survival metrics were calculated exclusively from records with complete information for all four reproductive and productive indicators. Prior to analysis, the database underwent quality control procedures to verify data completeness and internal consistency of birth and weaning records. Records presenting missing or incomplete information were excluded from the analyses and were not used to infer lamb mortality. Consequently, no imputation procedures were applied, and only complete records were considered for the calculation of survival-related outcomes, thereby minimizing potential misclassification bias due to administrative or recording errors. Descriptive statistics for the evaluated characteristics are presented in Table 2.

**Table 2:** Mean and standard deviation for total weight of progenies at birth (PWB) and weaning (PWW), and survival rate at birth (PSRB) and weaning (PSRW) in ewes of the Morada Nova breed

Traits	Average	Standard deviation
PWB (kg)	2.66	1.33
PWW (kg)	14.04	5.75
PSRB	0.85	0.34
PSRW	0.89	0.24

#### Statistical analysis

The statistical models were designed to account for the main biological, environmental, and management factors known to influence lamb growth and survival in semi-extensive sheep production systems.

For the adjustment of individual lamb weights at birth and weaning, linear models included the year of birth (1999–2015), and month of weighing (12 months). These factors were included to control for environmental variability associated with management differences among flocks and temporal fluctuations in climatic conditions typical of semiarid production systems. Parity order (1 to 8; with orders greater than 8 grouped) was included to account for maternal physiological maturity, as reproductive performance and maternal ability may change across successive lambings. Flock was included as a random effect to account for environmental and management variability among production units.

Sex of the progeny (male or female) was also included because sexual dimorphism in growth is well documented in sheep, particularly during early development. Additionally, the interaction between lamb sex composition and type of parity was considered in order to account for potential differences in intrauterine competition and maternal resource allocation among litter compositions (single male or female; twin births composed of two males, two females, or one male and one female; and triplets or higher-order multiple births regardless of sex).

Ewe body weight at lambing was included as a covariate because maternal body condition directly influences fetal development, milk production, and early lamb growth. For the adjustment of weaning weight, lamb age at weaning was also included as a covariate to account for variability in weaning timing under semi-extensive management conditions.

After adjusting individual lamb weights, the total progeny weight per ewe at birth (PWB) and at weaning (PWW) was calculated by summing the weights of lambs born or weaned per lambing event.

To evaluate the effect of prolificacy on productive traits (PWB and PWW), linear mixed models were fitted using least squares means, with type of parturition (single, double, and triple) included as a categorical fixed effect. In addition, regression models including the number of lambs per birth as a covariate were used to estimate the quantitative effect of each additional lamb on the studied traits. In these models, parity order, year, month of lambing, and ewe body weight at lambing were included as fixed effects to account for environmental, maternal, and management-related sources of variation. Pairwise comparisons among means were performed using the Tukey-Kramer test at a 5% significance level (*p* ≤ 0.05). Although type of parturition was used for categorical comparisons among groups, the number of lambs per birth was included as a continuous predictor in the mixed models to quantify the effect of prolificacy.

For survival traits at birth (PSRB) and at weaning (PSRW), generalized linear mixed models (GLMM) with binomial distribution and logit link function were fitted using individual lamb survival status (0 = dead, 1 = alive) as the response variable. These analyses were performed using the PROC GLIMMIX procedure of SAS (SAS Institute Inc., Cary, NC, USA). The models included type of parturition or number of lambs per birth as explanatory variables, along with the fixed effects of parity order, year, month of lambing, and ewe body weight at lambing.

The GLMM used for survival traits, assuming a binomial distribution with a logit link function, can be expressed as:

$$\begin{aligned}\mathrm{l}\mathrm{o}\mathrm{g}\mathrm{i}\mathrm{t}\left({\mathrm{p}}_{\mathrm{i}}\right)={\upmu\:}&+{{\upbeta\:}}_{1}\left({{\mathrm{n}}_{\mathrm{l}\mathrm{a}\mathrm{m}\mathrm{b}\mathrm{s}}}_{\mathrm{i}}\right)+{{\upbeta\:}}_{2}\left({\mathrm{p}\mathrm{a}\mathrm{r}\mathrm{i}\mathrm{t}\mathrm{y}}_{\mathrm{i}}\right)\cr &+{{\upbeta\:}}_{3}\left(\mathrm{e}\mathrm{w}{\mathrm{e}}_{{\mathrm{w}\mathrm{e}\mathrm{i}\mathrm{g}\mathrm{h}\mathrm{t}}_{\mathrm{i}}}\right)+{\mathrm{f}}_{\mathrm{l}}+{\mathrm{a}}_{\mathrm{m}},\end{aligned}$$

where $\:{\mathrm{p}}_{\mathrm{i}}$ is the probability of survival of lamb i; $\:{\upmu\:}$ is the overall mean; $\:{{\upbeta\:}}_{1}$, $\:{{\upbeta\:}}_{2}$ and $\:{{\upbeta\:}}_{3}$ are the regression coefficients associated with the fixed effects of number of lambs per birth, parity, and ewe weight; $\:{{\mathrm{n}}_{\mathrm{l}\mathrm{a}\mathrm{m}\mathrm{b}\mathrm{s}}}_{\mathrm{i}}$, $\:{\mathrm{p}\mathrm{a}\mathrm{r}\mathrm{i}\mathrm{t}\mathrm{y}}_{\mathrm{i}}$, and $\:\mathrm{e}\mathrm{w}{\mathrm{e}}_{{\mathrm{w}\mathrm{e}\mathrm{i}\mathrm{g}\mathrm{h}\mathrm{t}}_{\mathrm{i}}}$ are the observed covariates for lamb i; $\:{\mathrm{f}}_{\mathrm{l}}$ is the random effect of flock l ($\:{\mathrm{f}}_{\mathrm{l}}\sim\mathrm{N}(0,{{\upsigma\:}}_{\mathrm{f}\mathrm{l}\mathrm{o}\mathrm{c}\mathrm{k}}^{2})$); and $\:{\mathrm{a}}_{\mathrm{m}}$ is the random effect of ewe ($\:{\mathrm{a}}_{\mathrm{m}}\sim\mathrm{N}(0,{{\upsigma\:}}_{\mathrm{e}\mathrm{w}\mathrm{e}}^{2})$); in which $\:{{\upsigma\:}}_{\mathrm{f}\mathrm{l}\mathrm{o}\mathrm{c}\mathrm{k}}^{2}$ and $\:{{\upsigma\:}}_{\mathrm{e}\mathrm{w}\mathrm{e}}^{2}$ are the variances associated with flock and ewe effects, respectively.

Effect sizes from the GLMM were expressed as odds ratios (OR), obtained by exponentiating the model coefficients, and are interpreted as the multiplicative change in the odds of survival for a one-unit increase in the predictor. Corresponding 95% confidence intervals (CI) were also reported, representing the range of values within which the true parameter is expected to lie with 95% confidence.

To account for the hierarchical structure of the data and repeated lambings per ewe, ewe identification was included as a random effect. Flock was treated as a random effect because the objective was to make inferences beyond the specific flocks included in the study, considering them as a sample from a broader population of production systems in semiarid regions. This approach allows the partitioning of variability attributable to between-flock differences while preserving the generalizability of the results. The proportion of variance attributable to each hierarchical level was quantified using intraclass correlation coefficients (ICC). Variance components and ICC estimates were used to quantify the relative contribution of each hierarchical level (ewe and flock) to the total variability, providing additional insight into the structure of the data and supporting the interpretation of mixed model results.

The general linear mixed model used can be expressed as:

$$\begin{aligned}{\mathrm{y}}_{\mathrm{i}\mathrm{j}\mathrm{k}\mathrm{l}}={\upmu\:}&+{{{\upbeta\:}}_{1}\left({\mathrm{n}}_{\mathrm{l}\mathrm{a}\mathrm{m}\mathrm{b}\mathrm{s}}\right)}_{\mathrm{i}}+{{{\upbeta\:}}_{2}\left(\mathrm{p}\mathrm{a}\mathrm{r}\mathrm{i}\mathrm{t}\mathrm{y}\right)}_{\mathrm{j}}\cr &+{{{\upbeta\:}}_{3}\left(\mathrm{e}\mathrm{w}{\mathrm{e}}_{\mathrm{w}\mathrm{e}\mathrm{i}\mathrm{g}\mathrm{h}\mathrm{t}}\right)}_{\mathrm{k}}+{\mathrm{f}}_{\mathrm{l}}+{\mathrm{a}}_{\mathrm{m}}+{\mathrm{e}}_{\mathrm{i}\mathrm{j}\mathrm{k}\mathrm{l}},\end{aligned}$$

where $\:{\mathrm{y}}_{\mathrm{i}\mathrm{j}\mathrm{k}\mathrm{l}}$ is the phenotype (PWB or PWW); $\:{\upmu\:}$ is the overall mean; $\:{{\upbeta\:}}_{1}$, $\:{{\upbeta\:}}_{2}$ and $\:{{\upbeta\:}}_{3}$ are the regression coefficients associated with number of lambs per birth, parity, and ewe weight, respectively; $\:{\mathrm{n}}_{\mathrm{l}\mathrm{a}\mathrm{m}\mathrm{b}\mathrm{s}}$ is the number of lambs per birth; $\:\mathrm{p}\mathrm{a}\mathrm{r}\mathrm{i}\mathrm{t}\mathrm{y}$ is the order of parturition; $\:\mathrm{e}\mathrm{w}{\mathrm{e}}_{\mathrm{w}\mathrm{e}\mathrm{i}\mathrm{g}\mathrm{h}\mathrm{t}}$ is the body weight of the ewe at lambing; $\:{\mathrm{f}}_{\mathrm{l}}$ is the random effect of flock ($\:{\mathrm{f}}_{\mathrm{l}}\sim\mathrm{N}(0,{{\upsigma\:}}_{\mathrm{f}\mathrm{l}\mathrm{o}\mathrm{c}\mathrm{k}}^{2})$); $\:{\mathrm{a}}_{\mathrm{m}}$ is the random effect of ewe ($\:{\mathrm{a}}_{\mathrm{m}}\sim\mathrm{N}(0,{{\upsigma\:}}_{\mathrm{e}\mathrm{w}\mathrm{e}}^{2})$); and $\:{\mathrm{e}}_{\mathrm{i}\mathrm{j}\mathrm{k}\mathrm{l}}$ is the residual error ($\:{\mathrm{e}}_{\mathrm{i}\mathrm{j}\mathrm{k}\mathrm{l}}\sim\mathrm{N}(0,{{\upsigma\:}}_{\mathrm{e}}^{2})$); in which $\:{{\upsigma\:}}_{\mathrm{f}\mathrm{l}\mathrm{o}\mathrm{c}\mathrm{k}}^{2}$, $\:{{\upsigma\:}}_{\mathrm{e}\mathrm{w}\mathrm{e}}^{2}$ and $\:{{\upsigma\:}}_{\mathrm{e}}^{2}$ are the variances associated with flock, ewe, and residual effects.

The ICC for ewe and flock, respectively, were calculated as $\:{\mathrm{I}\mathrm{C}\mathrm{C}}_{\mathrm{e}\mathrm{w}\mathrm{e}}=\frac{{{\upsigma\:}}_{\mathrm{e}\mathrm{w}\mathrm{e}}^{2}}{({{\upsigma\:}}_{\mathrm{e}\mathrm{w}\mathrm{e}}^{2}+{{\upsigma\:}}_{\mathrm{f}\mathrm{l}\mathrm{o}\mathrm{c}\mathrm{k}}^{2}+{{\upsigma\:}}_{\mathrm{e}}^{2})}$, and $\:{\mathrm{I}\mathrm{C}\mathrm{C}}_{\mathrm{f}\mathrm{l}\mathrm{o}\mathrm{c}\mathrm{k}}=\frac{{{\upsigma\:}}_{\mathrm{f}\mathrm{l}\mathrm{o}\mathrm{c}\mathrm{k}}^{2}}{({{\upsigma\:}}_{\mathrm{e}\mathrm{w}\mathrm{e}}^{2}+{{\upsigma\:}}_{\mathrm{f}\mathrm{l}\mathrm{o}\mathrm{c}\mathrm{k}}^{2}+{{\upsigma\:}}_{\mathrm{e}}^{2})}$.

Model adequacy was evaluated through residual diagnostics and goodness-of-fit assessment. For GLMM analyses, overdispersion was evaluated based on the ratio between the Pearson chi-square statistic and the residual degrees of freedom, and influence diagnostics were inspected to identify potentially influential observations.

For linear mixed models, model assumptions were evaluated by inspecting residual normality using Q-Q plots and the Shapiro-Wilk test, homogeneity of variance using residual-versus-fitted plots and Levene’s test, and independence of residuals through temporal residual inspection and the Durbin-Watson statistic. Multicollinearity among predictors was assessed using variance inflation factors (VIF). When necessary, logarithmic, square-root, or Box-Cox transformations were applied, and model diagnostics were reassessed.

Canonical discriminant analysis (CDA) was applied to evaluate the multivariate capacity of productive and survival traits to discriminate among parturition types. Unlike the univariate comparisons and regression analyses, which quantify the magnitude of prolificacy effects on individual traits, CDA allows the simultaneous evaluation of multiple variables and identifies which combination of traits best differentiates biological groups. This approach provides a complementary multivariate perspective on whether distinct productive profiles are associated with different litter sizes.

A stepwise selection procedure based on the F-statistic was used to determine which variables contributed significantly to group discrimination. Variables were entered into the model when F-to-enter ≥ 3.84 and removed when F-to-remove ≤ 2.71. The candidate variables considered in the analysis were total progeny weight at birth (PWB), total progeny weight at weaning (PWW), progeny survival rate at birth (PSRB), and progeny survival rate at weaning (PSRW). The general CDA model was:

$$\:{\mathrm{Z}}_{\mathrm{n}}=\:\propto\:+\:{{\upbeta\:}}_{1}{\mathrm{X}}_{1}+{{\upbeta\:}}_{2}{\mathrm{X}}_{2}+\:\cdots\:+\:{{\upbeta\:}}_{\mathrm{n}}{\mathrm{X}}_{\mathrm{n}},$$

where $\:{\mathrm{Z}}_{\mathrm{n}}$ represents the canonical discriminant function (or canonical variable), defined as a linear combination of the original variables used to discriminate among groups; $\:\propto\:$ is the intercept; $\:{\mathrm{X}}_{\mathrm{i}}$ are the explanatory variables; $\:{{\upbeta\:}}_{\mathrm{i}}$ are the discriminant coefficients for each explanatory variable.

The discriminant power of the canonical functions was evaluated based on the percentage of explained variance, Wilks’ Lambda statistic, and standardized canonical coefficients. In addition to identifying the traits contributing most strongly to discrimination among birth types, this analysis also allowed assessment of the classification accuracy of individuals among groups, providing insight into whether different parturition types exhibit distinguishable multivariate productive patterns.

Finally, with the aim of analyzing whether there was an association between the type of lambing of the ewe (single - S, multiple - M) and its type of progeny - statistical techniques such as the Chi-Square test and Fisher’s exact test were used to verify a possible association of prolificacy between the type of lambing of the mother and her offspring by seasonality. Statistical analyses were performed using SAS 9.4 and SPSS 25 software.

## Results and discussion

Significant differences (*P* < 0.0001) were observed among the evaluated traits according to type of parturition (Table 3), with lambs born from twin parturitions exhibiting higher individual birth weight (3.54 kg) compared with those from single (2.22 kg) and triplet parturitions (2.91 kg) (*P* < 0.05). Although single births are generally expected to result in heavier lambs due to the absence of intrauterine competition, the inverse pattern observed in the present study likely reflects better body condition and nutritional status of the dams during late gestation, greater placental efficiency in twin pregnancies, and management practices that favor more productive and robust females.

**Table 3:** Least squares means for total progeny weight at birth (PWB) and weaning (PWW), and survival rates at birth (PSRB) and weaning (PSRW) by type of parturition in Morada Nova ewes

Type of parturition	PWB (kg)	PWW (kg)	PSRB	PSRW
Simple	2.22^c^	15.68^b^	88.0^a^	100.0^a^
Double	3.54^a^	19.00^a^	85.0^b^	81.0^b^
Triple	2.91^b^	18.30^a^	64.0^c^	78.0^b^

Means followed by different letters in the column differ statistically by the Tukey - Kramer test (*p* < 0.0001)

Survival rates (PSRB and PSRW) here are expressed as percentages calculated within each parturition type and rounded to the nearest whole number

Note: The 100% survival rate observed for single births reflects the subset of complete records used in the analysis and should be interpreted with caution

These results contrast with regional data reported by Silva Júnior et al., (2019) who described lower birth weights for Morada Nova lambs from single (1.58 kg) and twin (1.77 kg) parturitions, suggesting that improvements in management, more favorable environmental conditions, genetic selection effects, and temporal differences between datasets may explain the higher values observed in the present study. Although lamb survival was not directly evaluated in this specific comparison, birth weight is widely recognized as one of the main determinants of neonatal viability, with average survival rates for the Morada Nova breed ranging from 82.2% from birth to weaning and 66.4% from birth to one year of age (Facó et al. 2008), while survival rates around 72% to weaning have been reported for different sheep breeds (Dorset (D), Finnsheep (F), Rambouillet (R), Suffolk (S) and Targhee (T) (Rosati et al. 2002). These references support the biological relevance of the birth weight patterns observed here, without implying direct inference on survival outcomes in the present dataset.

Regarding prolificacy, ewes with single births showed lower PWB and PWW values but higher PSRB and PSRW values (Table 3), which aligns with the findings of Aguirre et al. (2017) and Duguma et al. (2002), who also observed that increasing the number of lambs per birth reduces survival rates but increases total lamb weight at weaning. This is expected, as the weight of a lamb from a single birth does not exceed the total weight of lambs from twin births, meaning that PWB and PWW are lower in single births. On the other hand, single-bearing ewes offer a better chance of progeny survival at birth and weaning, due to factors such as increased maternal care and greater milk availability. This is evidenced by higher progeny survival rates from single births compared to multiple births. Aguirre et al. (2017) reported survival rates of 69.2, 47.6, and 38.4% for lambs from single, double, and triple births respectively, further demonstrating that PSRW decreases as the number of lambs per birth increases. Conversely, twin births, when resulting in live births, provide a greater total weight of lambs at birth and weaning. Duguma et al. (2002), in their study of Merino ewes, also observed greater PWB and PWW values for ewes with multiple births.

This trade-off between litter size and individual lamb performance highlights that prolificacy cannot be interpreted in isolation, as the number of lambs per birth directly affects individual birth weight, which is a key determinant of neonatal survival and overall productive efficiency. The lamb’s weight directly affects its survival rate at birth and weaning as it reflects factors such as nutrition, development, maternal ability, and the health of both the lamb and its mother. Thus, low birth weight is one of the main causes of mortality at this stage (Nóbrega Junior et al. 2005). Therefore, effective nutritional and health management of ewes and lambs postpartum is important to reducing mortality and, consequently, increasing the number of lambs at weaning. The total weight of lambs per ewe at weaning is a key productivity indicator, influenced by factors such as fertility, prolificity, survival rate, and maternal ability (Amarilho-Silveira et al. 2017).

For the linear mixed model fitted for total progeny weight traits (PWB and PWW), variance components were estimated as 0.092 for ewe effects, 0.081 for flock effects, and 0.642 for residual variation, resulting in a total phenotypic variance of 0.815. The corresponding intraclass correlation coefficients, calculated as the proportion of each component relative to the total variance, indicated that 11.3% of the total variance was attributable to differences among ewes and 9.9% to differences among flocks, while the remaining 78.8% was associated with residual variation.

Low lamb survival rates, particularly as litter size increases, are often associated with environmental and physiological constraints typical of semiarid production system. In the GLMM analysis, each additional lamb per birth was associated with a reduction in the odds of lamb survival (OR = 0.56; 95% CI: 0.54–0.58; *p* < 0.001), and parity also showed a negative effect on survival probability (OR = 0.77; 95% CI: 0.75–0.80; *p* < 0.001). In the first weeks of life, lamb mortality is primarily linked to nutritional limitations (e.g., inadequate colostrum intake, starvation and/or hypoglycemia), environmental stressors (such as thermal extremes, hypothermia during nocturnal temperature drops, predation, or crushing), and health-related factors (including congenital defects, infections, or trauma) (Nóbrega Junior et al. 2005). Lambs born from twin births generally exhibit lower individual birth weights due to increased intrauterine nutrient competition, which begins during gestation and may persist through the pre-weaning period, thereby increasing neonatal vulnerability. Under semiarid conditions, these physiological constraints may be exacerbated by seasonal fluctuations in forage and water availability, reduced maternal body condition at late gestation, and greater thermal challenges, collectively contributing to higher mortality risk, as reported in regional studies (Pires et al. 2011; Nóbrega Junior et al. 2005).

In the linear mixed regression model for PWB and PWW, the number of lambs per birth used as a covariate indicated that each additional lamb increased total birth weight by 1.74 kg (95% CI: 1.68–1.81; *p* < 0.001) and total weaning weight by 3.51 kg (95% CI: 2.930–4.097; *p* < 0.001) (Table 4). From a biological perspective, these increments are meaningful, as they reflect higher total output per ewe; however, they occurred concomitantly with a reduction of 6% in birth survival rate and 17% in weaning survival rate, which substantially constrains the net productive benefit under semi-extensive conditions. In production systems typical of semiarid regions, where nutritional supply, climatic stressors, and labor inputs are limiting, such survival losses may partially offset the gains in total lamb weight, suggesting that the productive advantage of higher prolificacy may be constrained when additional management interventions are not implemented.

**Table 4:** Regression analysis of the number of progenies per lamb on total weight of progenies at birth (PWB) and weaning (PWW), and survival rates at birth (PSRB) and weaning (PSRW) in Morada Nova ewes

Traits	Regression Coefficient (b)	Standard error of b	*p*-value
PWB (kg)	1.74	0.03	< 0.0001
PWW (kg)	3.51	0.19	< 0.0001
PSRB	-6.00	1.00	< 0.0001
PSRW	-17.00	1.00	< 0.0001

Thus, rather than evaluating prolificacy in isolation, the results indicate that prolificacy should be interpreted as a conditional trait, whose productive advantage depends on the system’s capacity to sustain adequate lamb survival. From a strategic breeding and management standpoint, selection should prioritize females that combine moderate prolificacy with high lamb survival and maternal ability, as this balance is more likely to optimize overall flock efficiency under semi-extensive conditions. This approach moves beyond a simplistic emphasis on litter size and provides a more robust criterion for improving productivity in challenging environments.

**Fig. 1:**
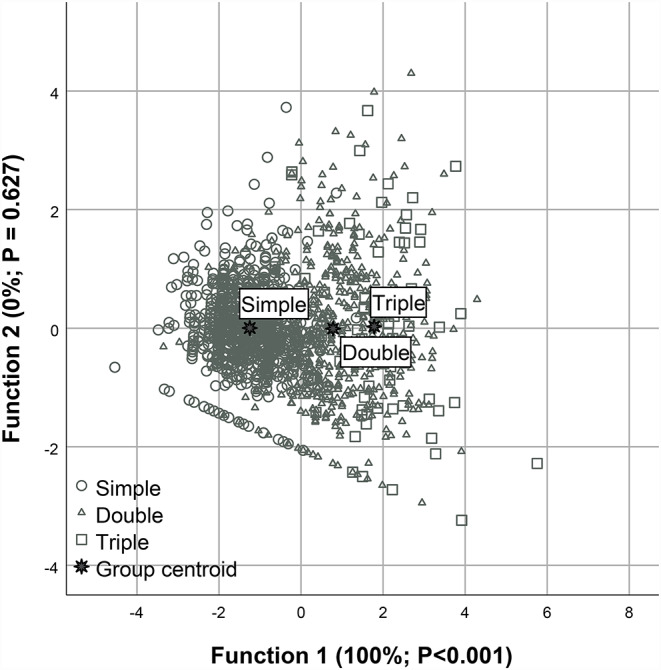
Canonical discriminant analysis biplot of productive indices of Morada Nova ewes according to parturition type (single, double and triple) in the Brazilian semiarid region. Note: Each point represents an individual ewe, and different symbols indicate parturition types. Function 1 explained virtually all of the total variation (100%; P < 0.001), clearly separating single from multiple births, whereas Function 2 accounted for a negligible proportion of the variation. Group centroids represent the multivariate mean of each parturition type

The summary of the canonical discriminant analysis considering the productive indices is presented in Table 5. In this multivariate approach, the candidate input variables were PWB, PWW, PSRB, PSRW. The first two canonical functions explained 100% of the total variation; however, only the first canonical function accounted for 99.9% of the variation and was statistically significant (*P* < 0.001). Among the evaluated variables, PWB was the only variable with sufficient discriminatory power to differentiate the birth types. The biplot of the two canonical functions and the classification rates shows this dynamic of birth weight as a function of the birth type, in which it is observed that the animals born from twin or triple births have similar weights (close centroids), but divergent from the single births (Fig. 1). Only 13.2% of the animals born from triple births were correctly classified in their group of origin, while the single birth animals presented a rate of 93.2%.

**Table 5:** Summary of canonical discriminant analysis for productive indices of Morada Nova sheep in the Brazilian semiarid region

**Eigenvalues**				
Function	Eigenvalues	% of variance	% cumulative	Canonical correlation
1	1.239^a^	100.0	100.0	0.744
2	0.000^a^	0.0	100.0	0.010

**Wilks’ Lambda**				
Test of functions	Wilks’ Lambda	Qui-square	df	*p*-value
1	0.446	1905.003	4	0.000
2	1.000	0.237	1	0.627
Functions	1		2	
PWB	0.98		-0.29	
PWW	0.1		1.01	

Classification results				
		Predict group association		
		Simple	Double	Triple
Count	Simple	932	78	0
	Double	188	931	33
	Triple	6	171	27
%	Simple	**92.3**	7.7	0.0
	Double	16.3	**80.8**	2.9
	Triple	2.9	83.8	**13.2**

Note:

Total weights of progenies at birth (PWB) and weaning (PWW)

Only 13.2% of lambs born from triplet births were correctly classified into their original group, whereas lambs from single births showed a markedly higher correct classification rate (93.2%). This extremely low discrimination accuracy for triplets indicates substantial overlap in the discriminant variables-particularly weight-related traits-between triplet-born lambs and those from other birth types. Such overlap suggests that, under the conditions evaluated, triplet lambs were unable to express a distinct phenotypic profile.

From a biological and production-system perspective, this pattern likely reflects nutritional and physiological constraints acting on high-order multiple pregnancies in semi-extensive systems. In triplet gestations, increased intrauterine competition for nutrients may reduce fetal growth and compress phenotypic variability, causing triplet lambs to resemble lighter individuals from single or twin births rather than forming a distinct multivariate group. This overlap likely explains the low discriminant separation observed for triplets, suggesting that under the studied conditions triplet births do not generate a clearly distinguishable productive phenotype.

The association tests between the ewe’s own birth type and the birth type of its progeny were not significant in either the dry or rainy seasons (Chi-square test: *p* = 0.55 and *p* = 0.11, respectively), indicating that the birth type of the ewe does not reliably predict the birth type of her offspring in this breed. From a biological and genetic standpoint, this lack of association suggests that prolificacy in Morada Nova sheep is likely governed by a complex interaction of polygenic effects, maternal physiological status, and environmental conditions, rather than being strongly inherited as a simple categorical trait linked to the ewe’s own birth type.

A statistically significant difference (*p* = 0.014) was detected between the average number of lambs weaned in the dry (1.65 ± 0.657) and rainy (1.60 ± 0.616) periods. However, despite statistical significance, the absolute difference between seasons was relatively small, suggesting limited biological magnitude under practical production conditions. Therefore, this result should be interpreted cautiously.

One possible explanation is related to the reproductive seasonality of the ewes. Lambings occurring during the dry season generally originate from conceptions during the preceding rainy season, when forage availability and maternal body condition are typically more favorable. Improved nutritional conditions during conception and early gestation may contribute to better reproductive outcomes (Daniel et al. 2013).

However, it is important to recognize that seasonal differences in reproductive performance may also be influenced by additional factors not fully captured in the dataset, including management practices, supplementation strategies, or variation in lambing supervision among flocks. Therefore, while seasonal nutritional dynamics may partially contribute to the observed pattern, the small magnitude of the difference suggests that multiple interacting factors likely influence the number of lambs weaned under semiarid production systems.

## Implications

Our results indicate that increasing prolificacy in Morada Nova ewes is associated with higher total lamb biomass at weaning but also with reduced progeny survival, particularly in multiple births. Under the semiarid conditions evaluated in this study, moderate prolificacy (one to two lambs per lambing) appeared to provide a more balanced outcome between productivity and survival. These findings highlight the importance of management strategies that ensure adequate nutritional support and careful monitoring of ewes carrying multiple fetuses, especially during late gestation and early lactation. From a breeding perspective, selection strategies that emphasize the number of lambs successfully weaned per ewe may be more suitable than those focusing solely on litter size. Future research could further explore the economic implications of different prolificacy levels in semiarid sheep production systems, particularly considering management costs and lamb survival rates.

## Conclusion

In Morada Nova ewes, multiple births increased productivity but reduced progeny survival, highlighting a clear trade-off between prolificacy and lamb viability. Moderate prolificacy proved to be more efficient for optimizing production under semiarid conditions, balancing total weaned lamb weight and survival rates. Furthermore, Morada Nova ewes tended to wean more lambs during the dry season than in the rainy season, reinforcing the influence of environmental seasonality on the breed’s productive and reproductive performance.

## Data Availability

Not applicable.

## Code availability

No applicable.
